# Relationship between Religious Orientation, Anxiety, and Depression among College Students: A Systematic Review and Meta-Analysis

**Published:** 2019-01

**Authors:** Sedighe FOROUHARI, Saeed HOSSEINI TESHNIZI, Mohammad Hasan EHRAMPOUSH, Seyed Saeed MAZLOOMY MAHMOODABAD, Hossein FALLAHZADEH, Seyed Ziaeddin TABEI, Mohammad NAMI, Masoud MIRZAEI, Bahia NAMAVAR JAHROMI, Seyyed Mehrdad HOSSEINI TESHNIZI, Jalil GHANI DEHKORDI, Maryamsadat KAZEMITABAEE

**Affiliations:** 1. Social Determinants of Health Research Center, Shahid Sadoughi University of Medical Sciences, Yazd, Iran; 2. Social Determinants in Health Promotion Research Center, Hormozgan University of Medical Sciences, Bandar Abbas, Iran; 3. Department of Environmental Health, School of Public Health, Shahid Sadoughi University of Medical Sciences, Yazd, Iran; 4. Prevention and Epidemiology of Non-Communicable Disease Research Center, Shahid Sadoughi University of Medical Sciences, Yazd, Iran; 5. Department of Medical Ethics and Philosophy of Health, School of Medicine, Shiraz University of Medical Sciences, Shiraz, Iran; 6. Department of Neuroscience, School of Advanced Medical Sciences and Technologies, Shiraz University of Medical Sciences, Shiraz, Iran; 7. Yazd Cardiovascular Research Center, Shahid Sadoughi University of Medical Sciences, Yazd, Iran; 8. Infertility Research Center, Shiraz University of Medical Sciences, Shiraz, Iran; 9. Department of Natural Science and Mathematics, School of Biochemistry, University of Texas at Dallas, Richardson, USA; 10. Social Determinants of Health Research Center, Shahrekord University of Medical Sciences, Shahrekord, Iran; 11. Isfahan Health Center, Isfahan University of Medical Sciences, Isfahan, Iran

**Keywords:** Religious orientation, Anxiety, Depression, Meta-analysis

## Abstract

**Background:**

Religious obligation helps people to develop mental health by creating internal commitment to special rules. This meta-analysis aimed to determine the relationship between religious orientation and anxiety among college students.

**Methods:**

Major scientific databases including PubMed, Web of Science, Science Direct, EBSCO, ProQuest and PsycINFO were searched for original research articles published 1987–2016. A random effect model was used to combine Correlation coefficient. All analyses were performed using Stata MP.

**Results:**

After screening of 7235 documents, 13 articles including 5620 participants met inclusion criteria in this meta-analysis. Correlation coefficient was −0.08 (95% CI= −0.19, −0.03) which indicated with increasing religious orientation, anxiety and depression reduced (*P*<0.001). Characteristics such as sex, geographic region, and type of religions were potential sources of heterogeneity. Based on fill-and-trim method the adjusted pooled r was obtained, −0.06 (95% CI= −0.16, −0.04).

**Conclusion:**

There was a weakness relationship between religious orientation and mental anxiety and depression. Therefore, it needs to improve knowledge of student about advantages of religious orientation.

## Introduction

Religion and faith refer to a set of customs and beliefs manifested in a religious entity (1). While it can be conceptualized as a wide structure without financial goals (2). Coning et al. (3), defined religion to facilitate movement toward God (4). Religion has a positive relationship with physical, mental, and physiological parameters (5, 6).

From religious perspective, there are internal and external religions. In internal religious orientation, faith is a transcendent value considered as inclusive motivation commitment (7). Moreover, internal religion is related to adaptation ability, expectancy (8), ability to find meaning of life in diseases (9), and better adaptation to stressful events (10). However, in external religious orientations, religion is an external tool to satisfy personal needs such as position and security. In other words, religiosity functions to obtain security and social status and those with this orientation, use religion as a tool to reach their wishes (11).

Most of mental disorders that result from psychological distress and bitterness of life are observed among non-religious people (12). Religious beliefs lead to positive emotions and feelings among people who improve physical health by strengthening the immune system (13). Since students constitute sensitive and important members of society with young age, their lifestyle is very important because it improves their health level where religion, is an important factor that promotes physical and mental health of this group (14).

According to the reports by National Mental Health Association (NMHA), 46% of male students and 64% of female students suffer from anxiety (3). The importance of anxiety and depression among students due to expansion of this disorder from initial stages of life (youth) to next stages, in addition to lowering daily activities, creates mental disorders, compliance problems, and crime in the future (6). Due to the importance that religion has in mental health of society, most medical schools in the United States (84 out of 126) are presenting religious courses (15). Studies on religious orientation among students with mental health indexes, such as depression and anxiety, have indicated different results for students in different countries (16, 17). Reviewing 130 studies, 34% of previous studies have pointed to a positive relationship between religious coping, adaptation, and mental health while 4% negatively evaluated this relationship and 62% of studies did not report any relationship between these variables (3, 8, 18). Moreover, by increasing religious orientation among students, depression and anxiety decreased and as a result, mental health increased (2).

Different educational, social, gender, racial, and cultural conditions of students in different countries can be the reasons for the controversial findings. To address this gap, the present study investigated the relationship between religion orientation and mental health among college students.

## Methods

### Ethic Committee Approval

We received the necessary permissions and obtaining the code of ethics with the characteristic IR.SSU.SPH.REC.1395.5 from the Ethics Committee of Shahid Sadoughi University of Medical Sciences for the necessary coordination.

### Search Strategy

This study was conducted according to PRISMA guidelines (http://www.prisma-statement.org/). The literature search was conducted via major online scientific databases including PubMed, Web of Science, Scopus and PsycInfo, EBSCO and ProQuest from Apr 1987 to Sep 2016 using the following keywords: “religion” OR “religiosity” OR “spirituality OR “religiousness” OR “Religious coping” OR ” Islam” OR “Mohammedanism” OR “Christianity” OR “church” OR “Religiousness” AND (“Orientation”) AND (“Anxiety” OR “Stress” OR “Depression “ OR “Mental health”) AND (“Student”).

### Inclusion and exclusion criteria

1. All studies included in this meta-analysis were required to have investigated the relationship between anxiety and religious orientation among student (undergraduate at university or high school) and excluded studies which had other participants.
2. Observational studies (cross-sectional, case-control) were included. Review articles and conference articles which not included quintile index (correlation coefficient) proceedings were excluded.
3. Studies with sample size more than 30 participates were included.
4. Language of studies was limited to English
5. The correlation coefficient between anxiety and religious orientation was reported, and sample size to calculate effect size (ES) was existed in the results of study.

### Data extraction

Information was extracted from the included studies by two independent reviewers (SF and SHT), who had experience with religious orientation and anxiety research and used a standard form to extract data. Any disagreement was resolved by discussion between the two reviewers. If consensus could not be reached, a third reviewer (EM) was consulted.

The standard form consisted of the following variables; title of study, authors’ names, year of publication, type of study, where the study was conducted, type of population, mean and standard deviation of correlation coefficient (19) age range (years), sample size, of study, sex, quality of study (QS) and the correlation coefficient between anxiety and religious orientation of student as a statistical index for using in meta-analysis. Quality of each included studies was assess by independent reviewers using standard checklist, Newcastle-Ottawa Scale (NOS). This tool has eight items and each item include three categories. The Kappa index used to examine agreement of two reviewers.

### Statistical analysis

In this meta-analysis, the correlation coefficient (r) was extracted from each study and then standard error (SE) was calculated by the following equation: $\sqrt{\frac{{\left(1-{\text{r}}^{2}\right)}^{2}}{\text{n}-1}}$ (where n is the sample size of study). Cochran’s heterogeneity statistic (*P*<0.1) and the I-squared index (25%: low; 50%: medium and 75%: high) were used to evaluate heterogeneity across effect sizes (ESs).

The results for each factor and pooled estimates were presented in a forest plot in which we reported the results as ES with 95% confidence intervals (CI). Meta-regression and subgroup analysis were used to evaluate source of heterogeneity among studies. Potential publication bias was explored using Funnel plot and Begg’s test. A trim-and-fill method was performed to detect the effect of missing studies on the overall effect of meta-analysis. All statistical analyses were done with the Statistical Software Package Stata MP version 14.

## Results

**Fig. 1:**
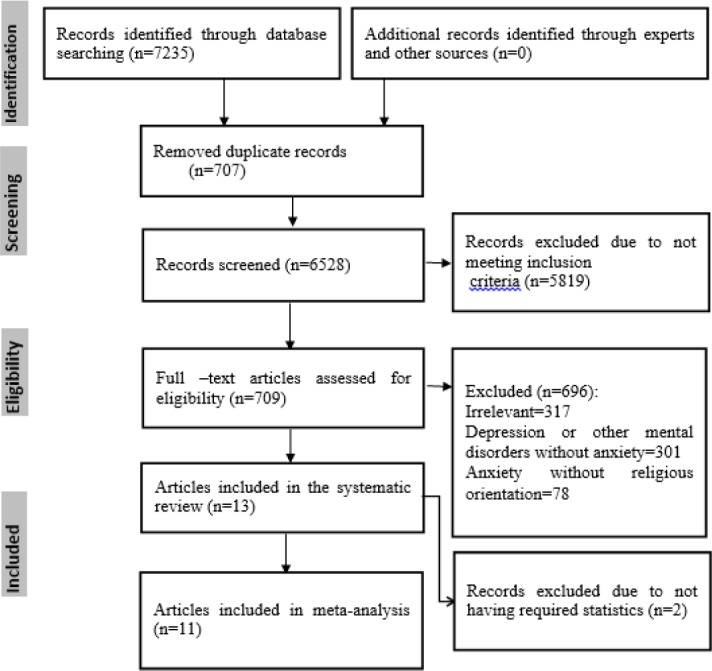
PRISMA flowchart describing the study design process

The search yielded 7244 articles of them 707 were duplicate records. After screening titles or abstracts by both authors, 5819 articles were removed as they did not meet all the inclusion criteria. Full text of 709 articles assessed for eligibility of them 696 records were excluded due to: being irrelevant (3176), reported depression, other mental disorders without anxiety (618) or anxiety without religious orientation (78 studies). Finally, 13 studies remained in the systematic review of which 11 studies reported correlation coefficient to be used in meta-analysis (Fig. 1).

Two reviewers assessed quality of included studies using a standard checklist; the Kappa index was examined to agreement of reviewers which showed a high agreement (Kappa=79%) between them. All included studies were cross-sectional study and most studies have been conducted in USA and Iran (%23), UK (%15) and Spain (%15). The total number of participants in the included studies was 5620 (range= 45 to 1786). Except for two studies, participates of other studies were undergraduate student at university (REFE). On average 54.1% of cases were male and the mean age of students was 19.9 yr. Six studies undertook on Muslims, two on Christian religions and three studies did not mention any particular religion.

In two studies, correlation coefficient was not reported, so were excluded (Table 1).

**Table 1:** The characteristics of selected studies for the meta-analysis of the relation between anxiety and religious orientation in college students 1986–2016

***Author***	***Year of study***	***Type of study***	***Country***	***Type of Population***	***Type of Religions***	***Total cases***	***Mean ±SD***	***Male (%)***	***QS***	***r***
Maltby J et al ( 1 )	1999	cross-sectional	UK	USU	Not reported	360	20.41±2.5 (18–29)	47.8	21	0.11
Amrai K et al ( 2 )	2011	cross-sectional	Iran	USU	Muslim	347	NR	38.9	23	NR
Buzdar M.A et al( 3 )	2014	cross-sectional	Iran	USU	Muslim	502	NR	0.0	18	0.011
García J et al( 4 )	2013	cross-sectional	Spain	USU	Non-Muslim	180	20.91±6.7 (18–55)	23.3	24	NR
Steffen P.R et al ( 5 )	2013	cross-sectional	USA	USU	Non-Muslim	1025	20.91±3.7 (18–31)	NR	17	−0.33
Lavrič M et al ( 6 )	2007	cross-sectional	UK	USU	Muslim and Non-Muslim	1786	20.91±1.5 (20–21)	NR	20	−0.149
Ghorbani N et al( 7 )	2008	cross-sectional	Iran	USU	Muslim	131	20.91±2.0 (NR)	38.9	25	−0.05
Khan Z.HP et al ( 8 )	2008	cross-sectional	Pakistan	USU	Muslim	160	21.4±1.8 (NR)	48.8	20	0.18
Bergin A et al ( 9 )	1987	cross-sectional		USU	No referred	151	NR	100.0	22	−0.27
Maltby, J., et al ( 10 )	1999	cross-sectional	Spain	USU	No referred	474	20.3±2.5 (18–29)	53.0	19	−0.17
Kuyel N et al ( 11 )	2012	cross-sectional	Turkish	USU	Muslim	341	21.05±1.6 (18–26)	100.0	14	−0.02
Davis T.L et al( 12 )	2003	cross-sectional	USA	HSS	Catholic, Protestant, Mormon, Jewish, other Christian, no religion	45	15.2±0.92 (14–17)	44.4	18	−0.45
Pierce Jr J.D et al( 13 )	2007	cross-sectional	USA	HSS and HSS	Catholic, Protestant	118	18.8±2.7 (13–25)	100.0	22	0.24

NR: Not Reported; USU: Undergraduate Student at University; HSS: High School Student; n: sample size; QS=Quality of studies

**Fig. 2:**
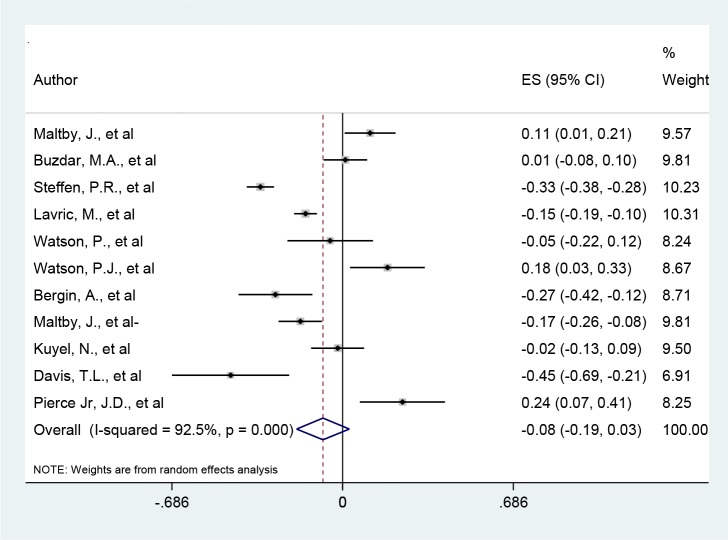
Forest plot of relationship between anxiety and religious orientation. The middle-point in each line indicates the correlation rate and the length of each line indicates the 95% confidence interval of each study. Diamonds indicate the 95% confidence interval for all studies

Generally, eleven studies met the eligibility criteria in this meta-analysis. The results of Q-Cochran and I-squared tests showed an evidence of high heterogeneity among correlation coefficient of studies (I-squared=92.5%, *P*<0.001). Using random effect meta-analysis pooled estimate of relationship between anxiety and religious orientation was −0.08 (95%CI=−0.19-0.03) which indicated that religious orientation was negatively correlated with anxiety in other words with increasing religious orientation, anxiety significantly reduces (*P*<0.001). In most studies (seven studies) a negative correlation between anxiety and religious orientation were reported; the highest and lowest correlation coefficients were reported, (r=−0.45) and (r=0.011) respectively (Fig. 2). To explore source of heterogeneity among the included studies meta-regression analysis was performed. Variables such as age, sex, country, type of religion, sample size and year of publication of paper were examined as possible sources of heterogeneity; however, the results of meta-regression indicated none of the variables may cause heterogeneity (*P*>0.05) ( Table 2 ).

**Table 2:** Meta-regression to assess characteristics effect on relationship between anxiety and religious

***Variables***	***Coeff.***	***Std. Err***	***t***	***P***
Age	0.11	0.06	2.06	0.18
Sex	0.0005	0.003	0.01	0.99
Country	−0.03	0.08	−0.40	0.73
Type of religions	0.29	0.23	1.25	0.34
Sample size	−0.0009	0.0007	0.01	0.99
Year of publication	0.04	0.02	1.64	0.24

In addition, subgroup analysis was used to identify the possible sources of heterogeneity. Inverse relationship between anxiety and religious orientation among females was larger than males. This relationship among students in the US was significantly larger than students in Europe and Asia. Moreover, non-Muslim students had significantly stronger correlation than Muslim and Non-Muslim students (*P*<0.001) (Table 3).

**Table 3:** Subgroup meta-analysis to compare relationship between anxiety and religious orientation

***Characteristics***	***Factors***	***N***	***r (95%CI)***	***I-square (%)***	***P-value***
Sex	Male	4	−0.06(−0.1, −0.03)	93.2	* P * <0.001
	Female	7	−0.22(−0.26, −0.18)	88.7	
Geographic region	America	3	−0.20(−0.26, 0.16)	96.5	* P * <0.001
	Europe	3	−0.14(−0.17, 0.10)	88.4	
	Asia	5	−0.008(−0.06, −0.04)	57.7	
Religion	Muslim	4	−0.01(−0.60, 0.42)	57.7	* P * <0.001
	Non-Muslim	4	−0.23(−0.27, −0.18)	96.3	
	Muslim and No-Muslim	3	−0.13(−0.16, −0.09)	88.8	

**Fig. 3:**
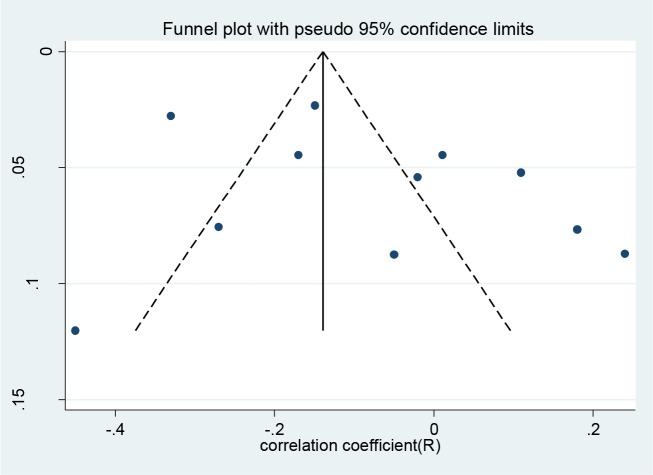
Funnel plot for publication bias among studies

The funnel plot for the included studies shows a relatively asymmetric. The results of Begg’s test confirmed these results (z=0.26, *P*=0.031), therefore, both methods indicated a considerable publication bias. After applying fill-and-trim method, the pooled estimate of relationship between anxiety and religious orientation found −0.060 (95% CI= −0.158, −0.038) and the pooled estimate found slightly larger. In other words, the effect of not included studies in meta-analysis found to be low (Fig. 3).

## Discussion

Religious orientation has a negative relationship with students’ anxiety. However, in external religion orientation, anxiety level increases. The inverse relationship between anxiety and religious orientation among women was more than men. These values were larger among Americans compared to Europeans and Asians, respectively. Moreover, non-Muslims had stronger correlation compared to Muslims and Christians, respectively. The relationship between religious orientation, mental health, and moral transformation was investigated among 384 students in Qom, Iran; mental health has positive relationship with religious orientation and moral transformation. In addition, religious orientation, gender, and education can predict mental health. By improving internal religious orientation and encouraging education, improving mental health is possible (20). Moreover, religious beliefs, PTSD, depression, and flexibility were investigated in survivors of earthquake in Haiti. About 92% of the participants believed in supernatural powers. There was a significant difference between decreased anxiety scores after the event, PTSD, and increased flexibility in people who believed these events are due to providence of God and those who had different views (21). Remembrance of God is a factor that increases tolerance against pressures that leads to increased self-control and moral development. With increased orientation toward internal religious, self-control ability increases that prevents the effects of external factors or demographic characteristics and preserves mental health. Moreover, performing religious orders and attending religious rituals can be effective in the treatment and prevention of mental diseases such as severe psychosis (22).

The results of a meta-analysis on religious studies and mental health showed that in 44% of studies, positive relationships are tangible and 23% showed negative relationships between religion and mental health and in 35% of studies, no significant relationship can be found (23).

It is possible to find evidence that indicate the positive effect of religion on mental health (24) which is consistent with the results of the present study, because the relationship between religious orientation and anxiety is stronger among Muslims and in Iran, as one of the Muslim countries, various studies have been conducted (25, 26) showed the effect of positive religious orientation on mental health.

A significant relationship was found between religious orientation and mental health dimensions where the highest correlation was observed between religious orientation, depression, and suicide. Whenever religious orientation decreases or becomes external, depression increases and intention to commit suicide increases, too. Religious people feel less depression with lower suicide intention. The relationship between uncontrollable stress and depression for people with external religious behavior was stronger compared to those with internal religious orientation (19). Therefore, people with internal religious orientation and belief in origin and destination of excellence are more hopeful, believe in God, predict pleasant events and in the case of unpleasant events, rely on God. The relationship between internal religious orientation and anxiety was observed (26) showed highest and lowest correlation coefficients, respectively. Evidence were observed based on religious humility and its acceptable correlation with depression symptoms at the level of two variables. By studying the obtained results based on the available evidence, this relationship is observed in both genders, age groups, and different ethnicities (27).

Conducted study on 140 male and female students studying at universities in Tehran, a direct relationship between religiosity and mental health was found. By increased external religious orientation, fatigue and physical symptoms increase. The highest correlation level was related to depression and intention toward suicide. External religious orientation was increasing parallel to increase in depression(23).

People’s health was investigated by scientific and religious views with hope, purpose in life, and fear of death among 474 Muslim students. Religiosity is positively related to all health factors. Scientific views were related to people’s health at higher levels. Hence, belief in science or God positively influences the health condition (28).

The relationship between religious orientation and depression were investigated among 571 students of Islamic Azad University of Azad Shahr. There are an inverse and significant relationship between religious orientation and depression and when students’ religious orientation increases, their depression and anxiety levels decrease. This study showed that there is a close relationship between students’ religious orientation, anxiety, and depression. Therefore, internalization of religious values can lead to increased mental health level of students (29). Despite contradictory and different definitions of religion and spirituality, both are considered as symbolic dimension of human that facilitate understanding of fundamental concepts such as purpose of creation and life. On the other hand, religion/spirituality are positively related to different mental and physiological health parameters. Religious beliefs can lead to positive emotions in people where improved immune system, promotes physical health.

Religion can create meaning; therefore, it gives meaning to life and death, increases life expectancy, and promotes optimism and can compensate decreased self-control. It prescribes a type of healthier lifestyle that positively influences mental health and includes a set of positive social norms that lead to acceptance by others, a type of supernatural feeling that undoubtedly causes psychological effects (30).

Limitations of this study consisted of low number of studies about variables of interest. Therefore, those studies that indirectly pointed to this issue such as studies on the relationship between anxiety, depression, and other psychological problems were used. Authors of this meta-analysis attempted to use all studies done at specific time intervals. Of course, there are different studies ignored by the authors of this study. Therefore, it is suggested to investigate those studies included non-student samples in future meta-analyses. There are numerous factors related to religious orientation and only limited variables are not sufficient to study the relationships and it is suggested to consider other variables in future studies. Such meta-analyses point to the gaps that exist in previous studies and help the author to consider the criteria ignored in previous studies. The limitations of such meta-analyses include access to sources and studies conducted and published in certain areas. Therefore, it is better to welcome repeated subjects to investigate more samples from the population of interest. Moreover, it rarely occurs that all studies which integrate with meta-analysis, show required indexes for analyses. This is considered among fundamental limitations of this meta-analysis.

## Conclusion

Religious teachings and improving beliefs help people to move toward perfection, growth, and mental health. Believe in God creates this ability in person to eliminate factors of anxiety and depression. According to the students’ mental health, universities and society should perform programs to improve religious values. Finally, despite lack of scientific interests in psychological dimension and humanities, religion can provide a framework for mental health. By providing the context and integration of religion with cognitive and emotional dimensions and moving from religious belief to internal religion, can improve mental health.

## Ethical considerations

Ethical issues (Including plagiarism, informed consent, misconduct, data fabrication and/or falsification, double publication and/or submission, redundancy, etc.) have been completely observed by the authors.
